# Vitamin A supplementation: who, when and how

**Published:** 2013

The majority of countries where vitamin A deficiency (VAD) is known to be a severe public health problem have policies supporting the distribution of vitamin A. This article provides guidelines for vitamin A supplementation in children and women and discusses when it is safe to phase out supplementation.

## Vitamin A supplements for young children aged 6–59 months

The World Health Organization (WHO) recommends that all children aged 6–59 months should receive supplements if they live in a community where VAD is a public health problem. These are communities where the prevalence of night blindness is ≥ 1% in children aged 24–59 months, or where the prevalence of VAD is ≥ 20% in infants and children aged 6–59 months.

The suggested vitamin A supplementation scheme for prevention of deficiency in children aged 6–59 months in areas where VAD is a severe public health problem is shown in Table 1.

## Vitamin A supplements for newborns and children aged 1–5 months

Vitamin A supplementation of newborns and children aged 1–5 months is not yet recommended by WHO. Exclusive breastfeeding of infants is encouraged for the first six months of life, to help achieve optimal growth, development and health.

## Vitamin A supplements for pregnant women are not routinely recommended

Although women are highly susceptible to VAD during pregnancy, vitamin A supplementation during pregnancy is not recommended, as high-dose vitamin A from supplements may cause harm to the developing baby. Instead, pregnant women are encouraged to meet their increased requirements by eating enough vitamin A-rich foods (see pages 65 and 72); this is unlikely to harm the developing foetus.

**Figure:**
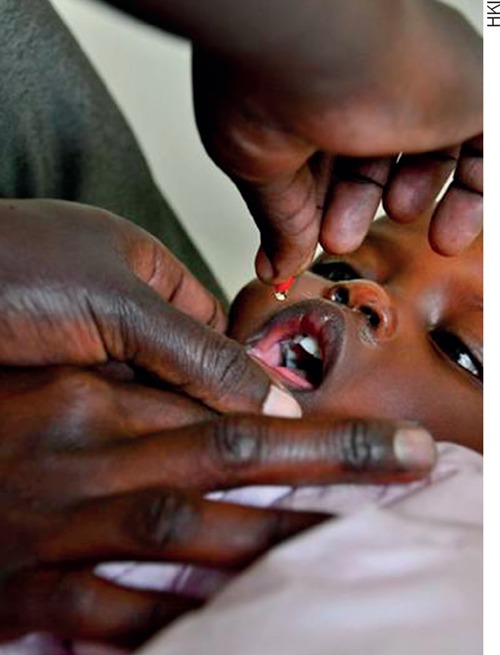
High-dose vitamin A in oral liquid form is given to a child

The **only** circumstance in which vitamin A supplementation during pregnancy may be considered is when women live in an area where VAD is a **severe public health problem** (i.e. ≥5% of pregnant women in that area have night blindness). It is very important to note that far lower doses are needed for pregnant women than for children, and doses need to be given on a more frequent basis (see Table 1).

## Vitamin A supplements for women who have recently given birth are not routinely recommended

Giving high-dose vitamin A to women immediately after delivery is also not recommended by the WHO (2011 Guidelines).

## When to phase out vitamin A supplements

WHO and the United Nations Children's Fund (UNICEF) recommend phasing out vitamin A supplementation when VAD is no longer a public health problem. This means there must be clear evidence that the prevalence of night blindness or reduced serum retinol levels are well below the minimum public health thresholds for an extended period of time and, at the same time, that mortality rates in under-5s are in long-term decline.

**Table:** Table 1. High-dose vitamin A supplementation to prevent deficiency in children aged 6–59 months

**Target age group**	**Oral dose**	**Frequency**	**Route of administration**
6–11 months	100,000 IU	Once	Oral liquid, oil-based preparation of retinyl palmitate or retinyl acetate
12–59 months	200,000 IU	Every 4–6 months	Oral liquid, oil-based preparation of retinyl palmitate or retinyl acetate

**Table:** Table 2. Low-dose vitamin A supplementation to prevent deficiency in pregnant women (*Note: ONLY in areas where vitamin A deficiency is a severe public health problem*)

**Target group**	**Oral dose**	**Frequency**	**Route of administration**	**Duration**
Pregnant women	Up to 10,000 IU vitamin A OR	Daily dose	Oral liquid, oil-based preparation of retinyl palmitate or retinyl acetate	A minimum of 12 weeks during pregnancy, until delivery
	Up to 25,000 IU vitamin A	Weekly dose

**Safety**

**Pregnant women**

Vitamin A supplements are not routinely recommended for pregnant women unless there is a severe public health problem. The far lower doses recommended in Table 2 are safe. Higher doses are contra-indicated because of uncertain effects on the unborn child.

**Children**

Vitamin A supplementation reduces child morbidity and mortality and is recommended for infants and children 6–59 months when VAD is a public health problem. Vitamin A supplements given to children will not cause any significant side effects when the recommended age-specific vitamin A dose is administered. Trials of vitamin A supplementation of infants and children aged 6–59 months have found uncommon, transient, and mild adverse symptoms (irritability, headache, fever, diarrhoea, nausea and vomiting). The impact of high-dose vitamin A supplements on preventing blindness and mortality, however, far outweigh these rare and transient side effects.
